# 4-(1,3-Benzothia­zol-2-yl)-*N*-(2-pyridylmeth­yl)aniline monohydrate

**DOI:** 10.1107/S1600536808041238

**Published:** 2008-12-10

**Authors:** Zhen-Hong Su, Qing-Zhi Wang, Lei Teng, Yong Zhang

**Affiliations:** a Medical School, Huangshi Institute of Technology, Huangshi 435003, People’s Republic of China; b School of Chemical and Materials Engineering, Huangshi Institute of Technology, Huangshi 435003, People’s Republic of China

## Abstract

In the title compound, C_19_H_15_N_3_S·H_2_O, the benzothia­zole ring system forms a dihedral angle of 7.22 (1)° with the benzene ring and the benzene ring forms a dihedral angle of 80.89 (1)° with the pyridine ring. An intra­molecular N—H⋯O inter­action is present. The crystal structure is stablized by inter­molecular O—H⋯N hydrogen bonds, π–π [centroid–centroid distances = 3.782 (1), 3.946 (1) and 3.913 (1) Å] and C—H⋯π inter­actions, forming a three dimensional-network.

## Related literature

For background information, see: Krebs *et al.* (2005 ▶); Kung *et al.* (2001 ▶); Naiki *et al.* (1989 ▶); Qu *et al.* (2007 ▶). For the synthetic procedure, see: Stephenson *et al.* (2007 ▶).
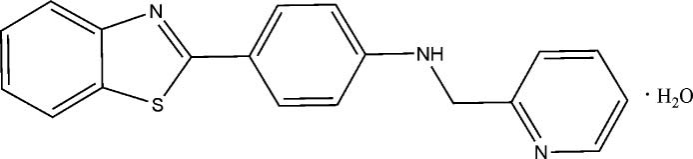


## Experimental

### 

#### Crystal data

C_19_H_15_N_3_S·H_2_O
*M* *_r_* = 335.42
Triclinic, 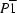

*a* = 6.5042 (3) Å
*b* = 11.5721 (5) Å
*c* = 11.9415 (5) Å
α = 99.597 (1)°
β = 103.599 (1)°
γ = 99.813 (1)°
*V* = 840.52 (6) Å^3^
*Z* = 2
Mo *K*α radiation
μ = 0.20 mm^−1^
*T* = 298 (2) K
0.20 × 0.10 × 0.10 mm

#### Data collection

Bruker SMART CCD diffractometer
Absorption correction: multi-scan (*SADABS*; Sheldrick, 1997 ▶) *T*_min_ = 0.961, *T*_max_ = 0.980
5408 measured reflections
3243 independent reflections
2342 reflections with *I* > 2σ(*I*)
*R*_int_ = 0.074

#### Refinement

*R*[*F* ^2^ > 2σ(*F* ^2^)] = 0.053
*wR*(*F* ^2^) = 0.133
*S* = 0.98
3243 reflections
227 parameters
4 restraints
H atoms treated by a mixture of independent and constrained refinement
Δρ_max_ = 0.25 e Å^−3^
Δρ_min_ = −0.28 e Å^−3^

### 

Data collection: *SMART* (Bruker, 2007 ▶); cell refinement: *SAINT-Plus* (Bruker, 2007 ▶); data reduction: *SAINT-Plus*; program(s) used to solve structure: *SHELXS97* (Sheldrick, 2008 ▶); program(s) used to refine structure: *SHELXL97* (Sheldrick, 2008 ▶); molecular graphics: *SHELXTL* (Sheldrick, 2008 ▶); software used to prepare material for publication: *SHELXTL*.

## Supplementary Material

Crystal structure: contains datablocks global, I. DOI: 10.1107/S1600536808041238/lh2740sup1.cif

Structure factors: contains datablocks I. DOI: 10.1107/S1600536808041238/lh2740Isup2.hkl

Additional supplementary materials:  crystallographic information; 3D view; checkCIF report

## Figures and Tables

**Table 1:** Hydrogen-bond geometry (Å, °)

*D*—H⋯*A*	*D*—H	H⋯*A*	*D*⋯*A*	*D*—H⋯*A*
N2—H2*A*⋯O1	0.856 (10)	2.030 (11)	2.877 (2)	171 (2)
O1—H1*B*⋯N1^i^	0.836 (9)	2.102 (11)	2.929 (2)	170 (3)
O1—H1*A*⋯N3^ii^	0.830 (9)	2.069 (10)	2.889 (2)	169 (3)
C18—H18⋯*Cg*^iii^	0.93	2.82	3.689 (3)	156

Symmetry codes: (i) 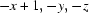; (ii) 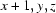; (iii) 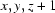.

**Fig. 1.**
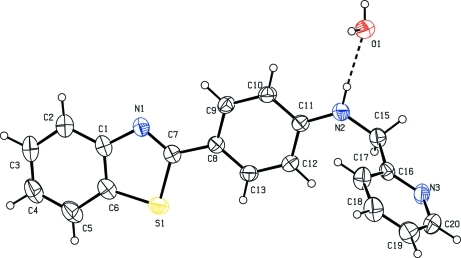
The molecular structure of (I), with displacement ellipsoids drawn at the 50% probability level. The dashed line indicates a hydrogen bond.

**Fig. 2.**
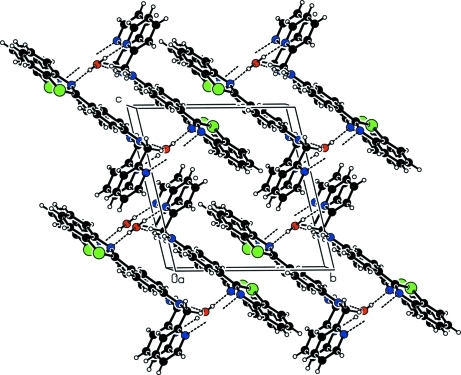
Part of the crystal structure showing H-bonds as dashed lines.

## supplementary crystallographic information

### Comment

Thioflavin T (ThT) is a benzothiazole dye that exhibits enhanced fluorescence upon binding to amyloid fibrils and is commonly used to diagnose these fibrils (Naiki *et al.*, 1989; Krebs *et al.*, 2005). In an effort to develop *in vivo* beta-sheet imaging probes, many derivatives of thioflavin T have been synthesized and evaluated (Kung *et al.*, 2001; Qu *et al.*, 2007). As part of our research, the title compound, (I), was prepared and we report the crystal stucture here.

The molecular structure is illustrated in Fig. 1. In (I), the benzothiazole unit is not coplanar with the benzene ring, forming a dihedral angle of 7.22 (1)°. The dihedral angle between the benzene ring and the pyridine ring is 80.89 (1)°. As shown in Fig. 2, molecules are linked into a three-dimensional network by a combination of N—H···O, O—H···N hydrogen bonds, C—H···π (Table 1) and π-π interactions. For the π-π interactions, some related parameters are listed as below: *Cg*1···*Cg*1^iv^ = 3.782 (1) Å, interplanar spacing: 3.680 (1) Å, dihedral angle: 0 °, symmetry code: iv) 1-*x*,1-y,-*z*; *Cg*2···*Cg*2^v^= 3.946 (1) Å, interplanar spacing: 3.678 (1) Å, dihedral angle: 0 °; symmetry code: v) –*x*,-y,1-*z*; *Cg*3···*Cg*^iv^ = 3.913 (1) Å, interplanar spacing: 3.748 (1) Å, dihedral angle: 7.1 (1)°. *Cg*1 is the centroid defined by atoms S1/N1/C1/C6/C7 while *Cg*2  and *Cg*3  are the centroids defined by atoms N3/C16—C20 and C1—C6.

### Experimental

Compound (I) was synthesized according to the method described by Stephenson *et al.* (2007). Yellow single crystals suitable for an X-ray diffraction study were obtained by slow evaporation of an ethanol solution.

### Refinement

H atoms bonded to C atoms were placed in idealized positions [*C*—*H*(methylene)=0.97 Å and C—H(aromatic) = 0.93 Å] and included in the refinement in the riding-motion approximation, with *U*_iso_=1.2*U*_eq_(C). H atoms bonded to N atoms and water O atoms were located in difference maps and then refined with the constraints of N-H = 0.86 (1)Å, O-H = 0.82 (1)Å and H-H = 1.35 (1)Å with *U*_iso_=1.2*U*_eq_(N) or 1.2*U*_eq_(O).

### Crystal data

**Table:**

C_19_H_15_N_3_S·H_2_O	*Z* = 2
*M**_r_* = 335.42	*F*(000) = 352
Triclinic, *P*1	*D*_x_ = 1.325 Mg m^−^^3^
Hall symbol: -P 1	Mo *K*α radiation, λ = 0.71073 Å
*a* = 6.5042 (3) Å	Cell parameters from 1809 reflections
*b* = 11.5721 (5) Å	θ = 1.8–26.0°
*c* = 11.9415 (5) Å	µ = 0.20 mm^−^^1^
α = 99.597 (1)°	*T* = 298 K
β = 103.599 (1)°	Plate, yellow
γ = 99.813 (1)°	0.20 × 0.10 × 0.10 mm
*V* = 840.52 (6) Å^3^	

### Data collection

**Table:**

Bruker SMART CCD diffractometer	3243 independent reflections
Radiation source: fine-focus sealed tube	2342 reflections with *I* > 2σ(*I*)
graphite	*R*_int_ = 0.074
φ and ω scans	θ_max_ = 26.0°, θ_min_ = 1.8°
Absorption correction: multi-scan (*SADABS*; Sheldrick, 1997)	*h* = −8→8
*T*_min_ = 0.961, *T*_max_ = 0.980	*k* = −11→14
5408 measured reflections	*l* = −14→10

### Refinement

**Table:**

Refinement on *F*^2^	Primary atom site location: structure-invariant direct methods
Least-squares matrix: full	Secondary atom site location: difference Fourier map
*R*[*F*^2^ > 2σ(*F*^2^)] = 0.053	Hydrogen site location: inferred from neighbouring sites
*wR*(*F*^2^) = 0.133	H atoms treated by a mixture of independent and constrained refinement
*S* = 0.98	*w* = 1/[σ^2^(*F*_o_^2^) + (0.0653*P*)^2^] where *P* = (*F*_o_^2^ + 2*F*_c_^2^)/3
3243 reflections	(Δ/σ)_max_ < 0.001
227 parameters	Δρ_max_ = 0.25 e Å^−^^3^
4 restraints	Δρ_min_ = −0.28 e Å^−^^3^

### Special details

**Table:**

Geometry. All e.s.d.'s (except the e.s.d. in the dihedral angle between two l.s. planes) are estimated using the full covariance matrix. The cell e.s.d.'s are taken into account individually in the estimation of e.s.d.'s in distances, angles and torsion angles; correlations between e.s.d.'s in cell parameters are only used when they are defined by crystal symmetry. An approximate (isotropic) treatment of cell e.s.d.'s is used for estimating e.s.d.'s involving l.s. planes.
Refinement. Refinement of *F*^2^ against ALL reflections. The weighted *R*-factor *wR* and goodness of fit *S* are based on *F*^2^, conventional *R*-factors *R* are based on *F*, with *F* set to zero for negative *F*^2^. The threshold expression of *F*^2^ > σ(*F*^2^) is used only for calculating *R*-factors(gt) *etc*. and is not relevant to the choice of reflections for refinement. *R*-factors based on *F*^2^ are statistically about twice as large as those based on *F*, and *R*- factors based on ALL data will be even larger.

### Fractional atomic coordinates and isotropic or equivalent isotropic displacement parameters (Å^2^)

**Table:**

	*x*	*y*	*z*	*U*_iso_*/*U*_eq_	
C1	0.4402 (3)	0.43406 (18)	−0.20560 (17)	0.0522 (5)	
C2	0.5812 (4)	0.4597 (2)	−0.2737 (2)	0.0691 (7)	
H2	0.6917	0.4186	−0.2762	0.083*	
C3	0.5534 (5)	0.5473 (2)	−0.3374 (2)	0.0801 (8)	
H3	0.6479	0.5659	−0.3826	0.096*	
C4	0.3900 (5)	0.6079 (2)	−0.3361 (2)	0.0845 (8)	
H4	0.3748	0.6659	−0.3810	0.101*	
C5	0.2492 (5)	0.5842 (2)	−0.2699 (2)	0.0785 (7)	
H5	0.1382	0.6252	−0.2692	0.094*	
C6	0.2765 (4)	0.49671 (19)	−0.20326 (18)	0.0580 (6)	
C7	0.2929 (3)	0.34399 (17)	−0.08419 (16)	0.0464 (5)	
C8	0.2525 (3)	0.26241 (17)	−0.00708 (16)	0.0463 (5)	
C9	0.3952 (3)	0.18903 (19)	0.02435 (17)	0.0517 (5)	
H9	0.5194	0.1946	−0.0020	0.062*	
C10	0.3564 (3)	0.10838 (19)	0.09370 (17)	0.0532 (5)	
H10	0.4541	0.0600	0.1128	0.064*	
C11	0.1720 (3)	0.09818 (18)	0.13580 (17)	0.0492 (5)	
C12	0.0305 (3)	0.17363 (18)	0.10587 (18)	0.0529 (5)	
H12	−0.0918	0.1701	0.1339	0.064*	
C13	0.0701 (3)	0.25266 (19)	0.03563 (18)	0.0528 (5)	
H13	−0.0275	0.3010	0.0160	0.063*	
C15	−0.0523 (3)	−0.00822 (19)	0.24393 (18)	0.0552 (5)	
H15A	−0.0695	−0.0890	0.2582	0.066*	
H15B	−0.1768	−0.0071	0.1811	0.066*	
C16	−0.0534 (3)	0.07696 (18)	0.35445 (16)	0.0479 (5)	
C17	0.1128 (4)	0.1740 (2)	0.41260 (19)	0.0659 (6)	
H17	0.2336	0.1912	0.3844	0.079*	
C18	0.0975 (5)	0.2455 (2)	0.5135 (2)	0.0775 (7)	
H18	0.2085	0.3115	0.5542	0.093*	
C19	−0.0808 (5)	0.2189 (2)	0.5533 (2)	0.0765 (7)	
H19	−0.0941	0.2657	0.6214	0.092*	
C20	−0.2392 (4)	0.1217 (2)	0.4904 (2)	0.0727 (7)	
H20	−0.3609	0.1032	0.5176	0.087*	
N1	0.4450 (3)	0.34715 (15)	−0.13801 (15)	0.0531 (4)	
N2	0.1390 (3)	0.01759 (17)	0.20410 (16)	0.0592 (5)	
H2A	0.228 (3)	−0.0288 (16)	0.2148 (19)	0.069 (7)*	
N3	−0.2300 (3)	0.05079 (16)	0.39105 (15)	0.0590 (5)	
O1	0.4135 (2)	−0.14685 (15)	0.26027 (16)	0.0699 (5)	
H1A	0.526 (3)	−0.0972 (19)	0.299 (2)	0.105*	
H1B	0.450 (4)	−0.2003 (18)	0.218 (2)	0.105*	
S1	0.12798 (10)	0.44713 (5)	−0.11189 (5)	0.0648 (2)	

### Atomic displacement parameters (Å^2^)

**Table:**

	*U*^11^	*U*^22^	*U*^33^	*U*^12^	*U*^13^	*U*^23^
C1	0.0604 (12)	0.0473 (12)	0.0409 (11)	0.0022 (10)	0.0057 (10)	0.0090 (9)
C2	0.0644 (14)	0.0747 (17)	0.0662 (15)	0.0035 (12)	0.0152 (12)	0.0250 (13)
C3	0.0883 (18)	0.0772 (18)	0.0708 (16)	−0.0063 (16)	0.0207 (14)	0.0314 (14)
C4	0.118 (2)	0.0620 (16)	0.0721 (17)	0.0093 (16)	0.0193 (17)	0.0320 (14)
C5	0.111 (2)	0.0603 (16)	0.0683 (16)	0.0275 (14)	0.0192 (15)	0.0222 (13)
C6	0.0778 (14)	0.0480 (12)	0.0430 (11)	0.0124 (11)	0.0102 (10)	0.0059 (10)
C7	0.0538 (11)	0.0440 (11)	0.0377 (10)	0.0095 (9)	0.0104 (9)	0.0024 (8)
C8	0.0536 (11)	0.0447 (11)	0.0393 (10)	0.0115 (9)	0.0126 (9)	0.0049 (9)
C9	0.0516 (11)	0.0609 (13)	0.0463 (11)	0.0149 (10)	0.0177 (9)	0.0129 (10)
C10	0.0599 (12)	0.0596 (13)	0.0478 (12)	0.0242 (10)	0.0183 (10)	0.0154 (10)
C11	0.0603 (12)	0.0486 (12)	0.0399 (10)	0.0139 (10)	0.0165 (9)	0.0069 (9)
C12	0.0582 (12)	0.0539 (13)	0.0538 (12)	0.0182 (10)	0.0253 (10)	0.0106 (10)
C13	0.0606 (12)	0.0497 (12)	0.0528 (12)	0.0207 (10)	0.0192 (10)	0.0098 (10)
C15	0.0672 (13)	0.0500 (12)	0.0495 (12)	0.0096 (10)	0.0204 (10)	0.0104 (10)
C16	0.0602 (12)	0.0452 (12)	0.0395 (10)	0.0091 (10)	0.0133 (9)	0.0149 (9)
C17	0.0731 (15)	0.0648 (15)	0.0502 (13)	−0.0061 (12)	0.0172 (11)	0.0067 (11)
C18	0.0994 (19)	0.0625 (16)	0.0526 (14)	−0.0088 (14)	0.0143 (14)	−0.0003 (12)
C19	0.120 (2)	0.0629 (16)	0.0481 (14)	0.0175 (16)	0.0324 (15)	0.0041 (12)
C20	0.0930 (18)	0.0738 (17)	0.0644 (15)	0.0196 (14)	0.0441 (14)	0.0167 (13)
N1	0.0573 (10)	0.0527 (10)	0.0500 (10)	0.0111 (8)	0.0145 (8)	0.0145 (8)
N2	0.0734 (12)	0.0598 (12)	0.0590 (11)	0.0262 (10)	0.0310 (10)	0.0225 (10)
N3	0.0709 (12)	0.0540 (11)	0.0558 (11)	0.0069 (9)	0.0288 (9)	0.0125 (9)
O1	0.0666 (10)	0.0648 (11)	0.0849 (12)	0.0193 (8)	0.0325 (9)	0.0125 (9)
S1	0.0899 (5)	0.0624 (4)	0.0565 (4)	0.0371 (3)	0.0298 (3)	0.0170 (3)

### Geometric parameters (Å, °)

**Table:**

C1—C2	1.389 (3)	C11—C12	1.400 (3)
C1—C6	1.390 (3)	C12—C13	1.372 (3)
C1—N1	1.390 (3)	C12—H12	0.9300
C2—C3	1.376 (3)	C13—H13	0.9300
C2—H2	0.9300	C15—N2	1.436 (3)
C3—C4	1.371 (4)	C15—C16	1.512 (3)
C3—H3	0.9300	C15—H15A	0.9700
C4—C5	1.366 (4)	C15—H15B	0.9700
C4—H4	0.9300	C16—N3	1.328 (2)
C5—C6	1.398 (3)	C16—C17	1.373 (3)
C5—H5	0.9300	C17—C18	1.378 (3)
C6—S1	1.723 (2)	C17—H17	0.9300
C7—N1	1.299 (3)	C18—C19	1.361 (4)
C7—C8	1.458 (3)	C18—H18	0.9300
C7—S1	1.757 (2)	C19—C20	1.360 (3)
C8—C9	1.391 (3)	C19—H19	0.9300
C8—C13	1.392 (3)	C20—N3	1.345 (3)
C9—C10	1.377 (3)	C20—H20	0.9300
C9—H9	0.9300	N2—O1	2.877 (2)
C10—C11	1.400 (3)	N2—H2A	0.856 (10)
C10—H10	0.9300	O1—H1A	0.830 (9)
C11—N2	1.360 (3)	O1—H1B	0.836 (9)
			
C2—C1—C6	119.7 (2)	C12—C13—C8	121.7 (2)
C2—C1—N1	125.2 (2)	C12—C13—H13	119.1
C6—C1—N1	115.02 (19)	C8—C13—H13	119.1
C3—C2—C1	118.4 (2)	N2—C15—C16	115.24 (17)
C3—C2—H2	120.8	N2—C15—H15A	108.5
C1—C2—H2	120.8	C16—C15—H15A	108.5
C4—C3—C2	121.8 (3)	N2—C15—H15B	108.5
C4—C3—H3	119.1	C16—C15—H15B	108.5
C2—C3—H3	119.1	H15A—C15—H15B	107.5
C5—C4—C3	120.9 (3)	N3—C16—C17	122.3 (2)
C5—C4—H4	119.5	N3—C16—C15	114.46 (18)
C3—C4—H4	119.5	C17—C16—C15	123.3 (2)
C4—C5—C6	118.2 (3)	C16—C17—C18	118.9 (2)
C4—C5—H5	120.9	C16—C17—H17	120.6
C6—C5—H5	120.9	C18—C17—H17	120.6
C1—C6—C5	121.0 (2)	C19—C18—C17	119.6 (2)
C1—C6—S1	109.75 (17)	C19—C18—H18	120.2
C5—C6—S1	129.3 (2)	C17—C18—H18	120.2
N1—C7—C8	124.97 (18)	C20—C19—C18	118.0 (2)
N1—C7—S1	114.73 (15)	C20—C19—H19	121.0
C8—C7—S1	120.30 (15)	C18—C19—H19	121.0
C9—C8—C13	117.53 (19)	N3—C20—C19	123.8 (2)
C9—C8—C7	120.40 (18)	N3—C20—H20	118.1
C13—C8—C7	122.05 (19)	C19—C20—H20	118.1
C10—C9—C8	121.39 (19)	C7—N1—C1	111.11 (18)
C10—C9—H9	119.3	C11—N2—C15	124.34 (19)
C8—C9—H9	119.3	C11—N2—O1	124.36 (14)
C9—C10—C11	120.9 (2)	C15—N2—O1	110.78 (13)
C9—C10—H10	119.5	C11—N2—H2A	117.8 (16)
C11—C10—H10	119.5	C15—N2—H2A	117.1 (16)
N2—C11—C12	123.07 (19)	C16—N3—C20	117.4 (2)
N2—C11—C10	119.30 (19)	N2—O1—H1A	99 (2)
C12—C11—C10	117.63 (19)	N2—O1—H1B	132 (2)
C13—C12—C11	120.76 (19)	H1A—O1—H1B	107.1 (15)
C13—C12—H12	119.6	C6—S1—C7	89.37 (10)
C11—C12—H12	119.6		
			
C6—C1—C2—C3	0.0 (3)	N2—C15—C16—N3	−179.05 (18)
N1—C1—C2—C3	179.0 (2)	N2—C15—C16—C17	1.3 (3)
C1—C2—C3—C4	−0.8 (4)	N3—C16—C17—C18	1.1 (4)
C2—C3—C4—C5	0.8 (4)	C15—C16—C17—C18	−179.2 (2)
C3—C4—C5—C6	0.1 (4)	C16—C17—C18—C19	−0.1 (4)
C2—C1—C6—C5	0.9 (3)	C17—C18—C19—C20	−0.3 (4)
N1—C1—C6—C5	−178.17 (19)	C18—C19—C20—N3	−0.3 (4)
C2—C1—C6—S1	−179.81 (16)	C8—C7—N1—C1	179.53 (17)
N1—C1—C6—S1	1.1 (2)	S1—C7—N1—C1	0.3 (2)
C4—C5—C6—C1	−1.0 (3)	C2—C1—N1—C7	−179.93 (18)
C4—C5—C6—S1	179.93 (19)	C6—C1—N1—C7	−0.9 (2)
N1—C7—C8—C9	7.2 (3)	C12—C11—N2—C15	6.3 (3)
S1—C7—C8—C9	−173.62 (15)	C10—C11—N2—C15	−174.45 (18)
N1—C7—C8—C13	−171.42 (18)	C12—C11—N2—O1	177.22 (15)
S1—C7—C8—C13	7.8 (3)	C10—C11—N2—O1	−3.5 (3)
C13—C8—C9—C10	0.9 (3)	C16—C15—N2—C11	−84.0 (3)
C7—C8—C9—C10	−177.73 (18)	C16—C15—N2—O1	104.04 (17)
C8—C9—C10—C11	−0.5 (3)	C17—C16—N3—C20	−1.7 (3)
C9—C10—C11—N2	−179.97 (19)	C15—C16—N3—C20	178.7 (2)
C9—C10—C11—C12	−0.7 (3)	C19—C20—N3—C16	1.3 (4)
N2—C11—C12—C13	−179.35 (19)	C1—C6—S1—C7	−0.75 (15)
C10—C11—C12—C13	1.4 (3)	C5—C6—S1—C7	178.4 (2)
C11—C12—C13—C8	−1.0 (3)	N1—C7—S1—C6	0.28 (16)
C9—C8—C13—C12	−0.2 (3)	C8—C7—S1—C6	−179.01 (16)
C7—C8—C13—C12	178.42 (18)		

### Hydrogen-bond geometry (Å, °)

**Table:**

*D*—H···*A*	*D*—H	H···*A*	*D*···*A*	*D*—H···*A*
N2—H2A···O1	0.86 (1)	2.03 (1)	2.877 (2)	171 (2)
O1—H1B···N1^i^	0.84 (1)	2.10 (1)	2.929 (2)	170 (3)
O1—H1A···N3^ii^	0.83 (1)	2.07 (1)	2.889 (2)	169 (3)
C18—H18···Cg^iii^	0.93	2.82	3.689 (3)	156

Symmetry codes: (i) −*x*+1, −*y*, −*z*; (ii) *x*+1, *y*, *z*; (iii) *x*, *y*, *z*+1.
